# Abortive Infection of Snakehead Fish Vesiculovirus in ZF4 Cells Was Associated with the RLRs Pathway Activation by Viral Replicative Intermediates

**DOI:** 10.3390/ijms16036235

**Published:** 2015-03-18

**Authors:** Wenwen Wang, Muhammad Asim, Lizhu Yi, Abeer M. Hegazy, Xianqin Hu, Yang Zhou, Taoshan Ai, Li Lin

**Affiliations:** 1 Department of Aquatic Animal Medicine, College of Fisheries, Huazhong Agricultural University, Wuhan 430070, China; E-Mails: wangwenwen1030@webmail.hzau.edu.cn (W.W.); asim_m97@yahoo.com (M.A.); vanessa0324@webmail.hzau.edu.cn (L.Y.); abeerhejazy@gmail.com (A.M.H.); xianqinhu@webmail.hzau.edu.cn (X.H.); zhouyang@mail.hzau.edu.cn (Y.Z.); 2 Freshwater Aquaculture Collaborative Innovation Center of Hubei Province, Wuhan 430070, China; 3 Central Laboratory for Environmental Quality Monitoring (CLEQM), National Water Research Center (NWRC), El-Kanater 13621, Egypt; 4 School of Animal Science and Nutritional Engineering, Wuhan Polytechnic University, Wuhan 430023, China; 5 Wuhan Fishery Research Institute, Wuhan 430207, China; E-Mail: ats888@126.com; 6 Key Lab of Freshwater Animal Breeding, Ministry of Agriculture, Wuhan 430070, China

**Keywords:** snakehead fish vesiculovirus, ZF4, infection, Retinoic acid-inducible gene I (RIG-I)-like receptors, interferon, viral replicative intermediates

## Abstract

Snakehead fish vesiculovirus (SHVV) is a negative strand RNA virus which can cause great economic losses in fish culture. To facilitate the study of SHVV-host interactions, the susceptibility of zebrafish embryonic fibroblast cell line (ZF4) to the SHVV was investigated in this report. The results showed that high amount of viral mRNAs and cRNAs were detected at the 3 h post-infection. However, the expressions of the viral mRNAs and cRNA were decreased dramatically after 6 h post-infection. In addition, the expressions of interferon (IFN) and interferon-induced GTP-binding protein Mx were all up regulated significantly at the late stage of the infection. Meanwhile, the expressions of Retinoic acid-inducible gene I (RIG-I) and Melanoma differentiation-associated gene 5 (MDA5) were also all up-regulated significantly during the infection. Two isoforms of DrLGP2 from zebrafish were also cloned and analyzed. Interestingly, the expression of DrLGP2a but not DrLGP2b was significantly up-regulated at both mRNA and protein levels, indicating that the two DrLGP2 isoforms might play different roles during the SHVV infection. Transfection experiment showed that viral replicative intermediates were required for the activation of IFN-α expression. Taken together, the abortive infection of SHVV in ZF4 cells was associated with the activation of RLRs pathway, which was activated by viral replicative intermediates.

## 1. Introduction

RIG-I-like receptors (RLRs) are the members of pattern recognition receptors (PRRs) which are critical for triggering the downstream NF-κB pathway, resulting in the production of type I interferons (IFNs) during the virus infection. There are three members of RLRs, named Retinoic acid-inducible gene 1 (RIG-I), Melanoma differentiation-associated gene 5 (MDA5), and Laboratory of genetics and physiology 2 (LGP2) [1]. All RLRs share homologous core domains, including a central DEXD/H helicase domain and a carboxyterminal regulatory domain (RD) [1]. RIG-I and MDA5 contain additional caspase activation and recruitment domains (CARDs) at the *N*-terminus, while LGP2 has no CARDs [1,2]. Since RIG-I and MDA5 are able to induce cellular immune responses upon recognition of viral double-stranded RNA (dsRNA) via CARDs [3,4,5,6,7]. However, LGP2 is not able to induce signaling alone due to the lack of CARDs at *N*-terminal region. In teleost, RLRs have been reported in many fish, such as Zebrafish [8,9,10,11,12], Rainbow trout [13], Atlantic salmon [14], Grass carp [15,16,17] and Japanese flounder [18]. There is growing evidence that fish RIG-I and MDA5 can also activate the interferon response via a mitochondrion associated signaling pathway [8,9,10,11,12,13,19]. Recently, the roles of RLRs from rainbow trout (*Oncorhynchus mykiss*) have been investigated. Two LGP2 isoforms were identified from rainbow trout, named LGP2a and LGP2b (with a deletion of 54 amino acid residues at the *C*-terminus) [13].

Snakehead fish vesiculovirus (SHVV) is a new member of the family *Rhabdoviridae* which could cause great economic losses in snakehead fish culture [19]. The genome of SHVV consists of a negative, single-stranded RNA which encodes five viral structural proteins, including Nucleoprotein (N), Phosphoprotein (P), Matrix protein (M), Glycoprotein (G), large protein (L) or RNA dependent RNA polymerase [19]. Recently, we showed that Mandarin fish was susceptible to SHVV, indicating that SHVV might have broad host ranges [20]. Zebrafish (*Danio rerio*) has become an attractive research model for the studies of fish diseases. Zebra fish embryonic fibroblast cell line (ZF4) is a common cell line which is widely used in the labs [21]. To study the virus-host interactions during the SHVV infection, we included ZF4 for our virus infection experiments.

## 2. Results

### 2.1. Characterization of DrLGP2a and DrLGP2b cDNAs

The full-length DrLGP2a and DrLGP2b cDNAs were deposited in GenBank (accession numbers; KP 341002 and KP341003). The DrLGP2b was a truncated isoform of DrLGP2a with the deletion at the *C*-terminal at 525 aa. The complete sequences of DrLGP2a cDNA consisted of a 5'-terminal untranslated region (UTR) of 50 bp, a 3'-UTR of 383 bp with a poly (A) tail, and an open reading frame (ORF) of 2040 bp (Supplementary Figure S1). The ORF encoded a polypeptide of 679 amino acids with an isoelectric point of 8.24 and predicted molecular weight of 77.76 kD. The complete sequences of DrLGP2b cDNA consisted of a 5'-UTR of 50 bp, a 3'-UTR of 641 bp with a poly (A) tail, and an open reading frame (ORF) of 1575 bp (Supplementary Figure S2). The ORF encoded a polypeptide of 524 amino acids with an isoelectric point of 6.78 and predicted molecular weight of 59.49 kD. The typical polyadenylation signal AATAAA was not found in the DrLGP2a mRNA, but it was present in DrLGP2b mRNA. As shown in Supplementary Figure S3, DrLGP2a and DrLGP2b both contain an *N*-terminal ATP dependent DEXDc domain (1–220 aa), an RNA helicase HELICc domain (HELICc) (341–485 aa) which were also present in RIG-I and MDA5.Compare with DrLGP2a, the DrLGP2b lack a regulatory domin (RD) (551–672 aa) at the *C*-terminal.

**Figure 1:**
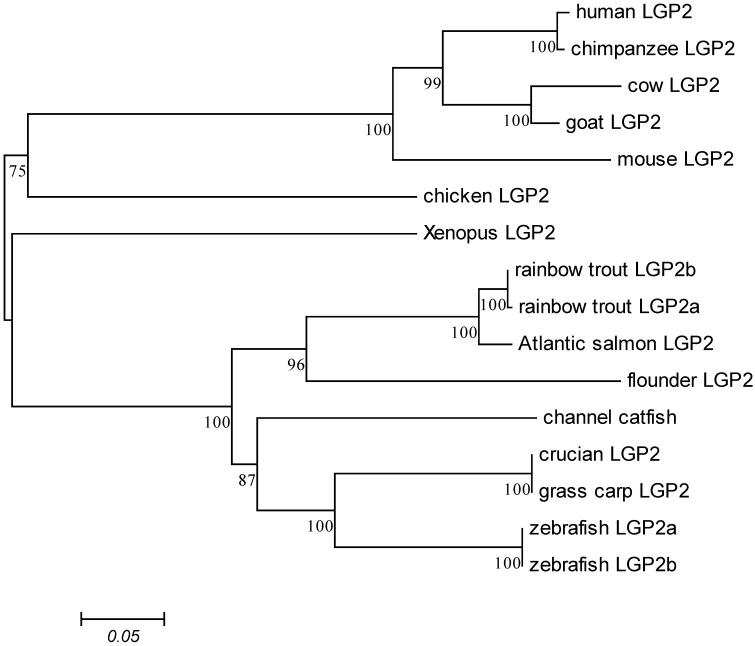
Phylogenetic tree of the laboratory of genetics and physiology 2 (LGP2) from zebrafish and other species. The protein sequences used for phylogenetic analysis were Atlantic salmon LGP2 (*Salmo salar*, ACI33640.1), channel catfish (*Ictalurus punctatus*, AFS34610.1), chicken LGP2 (*Gallus gallus*, AEK21509.1), chimpanzee LGP2 (*Pan troglodytes*, JAA34322.1), cow LGP2 (*Bos taurus*, AAI46129.1), *crucian* LGP2 (Carassius auratus, AEN04474.1), flounder LGP2 (*Paralichthys olivaceus*, ADM18136.1), goat LGP2 (*Capra hircus*, XP_005693898.1), grass carp LGP2 (*Ctenopharyngodon idella*, AFQ93565.1), human LGP2 (*Homo sapiens*, NM_024119.2), mouse LGP2 (*Mus musculus*, NM_030150.2), rainbow trout LGP2a (*Oncorhynchus mykiss* splicevariant 1, CAZ27718.1), rainbow trout LGP2b (*Oncorhynchus mykiss* splice variant 2, CAZ27720.1), Xenopus LGP2 (*Xenopus laevis*, NP_001085915.1), zebrafish LGP2a (*Danio rerio*, KP341002), zebrafish LGP2b (*Danio rerio*, KP341003).

The phylogenetic tree was constructed using the LGP2 sequences from 14 taxa, including 6 teleosts, 1 amphibian, 1 avian and 6 mammals. The results clearly showed that *LGP2* genes formed teleost, amphibian, avian and mammal clades. LGP2 from zebrafish, Grass carp (*Ctenopharyngodon idella*) and crucian carp (*Carassius auratus*) were clustered together. Subsequently, they formed a teleost clade with LGP2 from channel catfish (*Ictalurus punctatus*), Japanese flounder (*Paralichthys olivaceus*), Atlantic salmon (*Salmo salar*) and rainbow trout (*Oncorhynchus mykiss*). Finally, the clade from fish was clustered with amphibian, avian and mammalian clades successively (Figure 1).

### 2.2. The mRNA and cRNA Expressions of the Viral N and G Genes in ZF4 Cells Infected with SHVV

**Figure 2:**
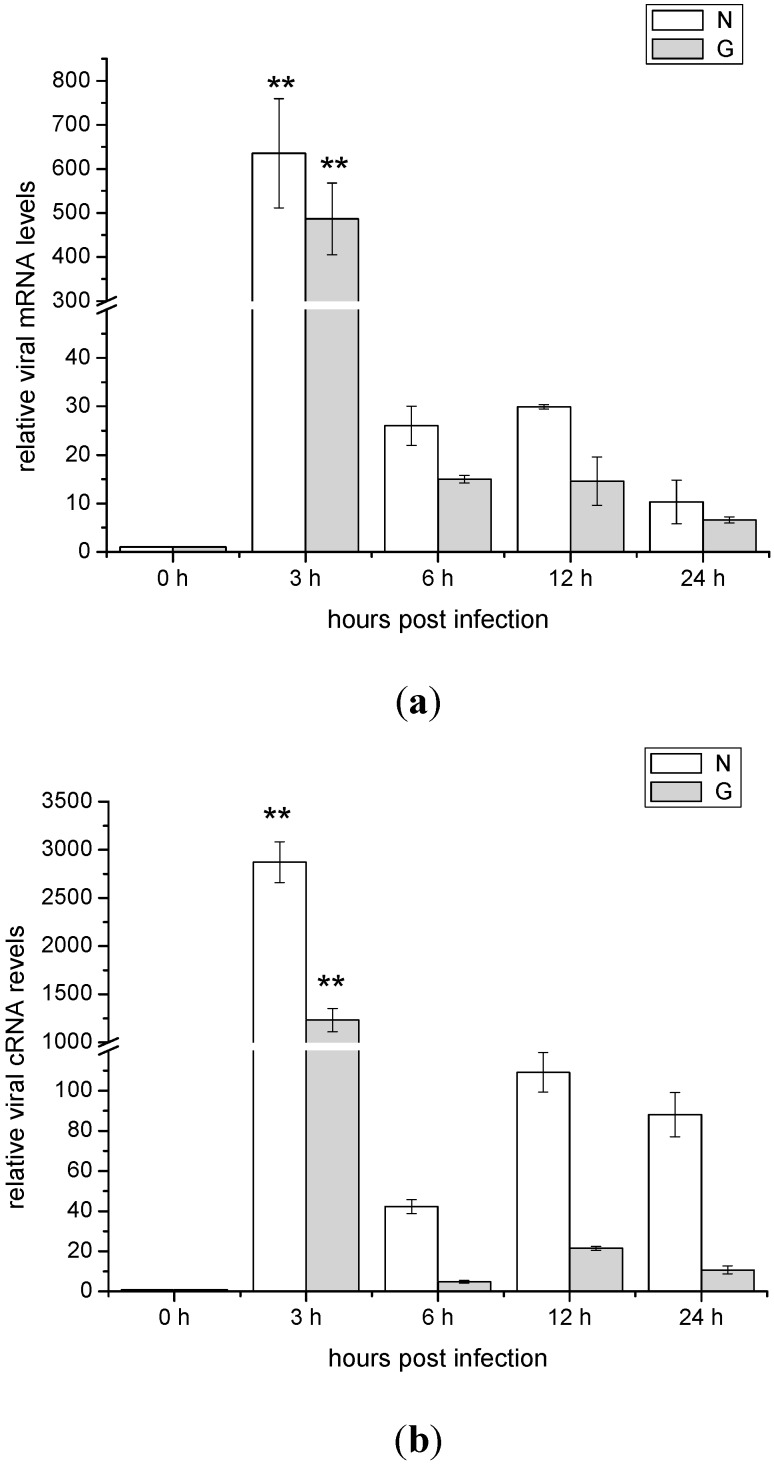
(**a**) Expression of viral N (Nucleoprotein) and G (Glycoprotein) genes mRNA in zebrafish embryonic fibroblast cell line (ZF4) with time after snakehead fish vesiculovirus (SHVV) infection. Expression was determined by qRT-PCR and β-actin was used as an internal control. Data were shown as mean ± SE (*n* = 3). The data were submitted to one-way analysis of variance (one-way ANOVA) followed by Fisher’s LSD test using SPSS. The asterisk indicated a statistically significant difference (** *p* < 0.01) compared with 0 h (set as 1); and (**b**) Expression of viral N and G genes cRNA in ZF4 with time after SHVV infection. Expression was determined by qRT-PCR and β-actin was used as an internal control. Data were shown as mean ± SE (*n* = 3). The data were submitted to one-way analysis of variance (one-way ANOVA) followed by Fisher’s LSD test using SPSS. The asterisk indicated a statistically significant difference (** *p* < 0.01) compared with 0 h (set as 1).

To monitor the replication of the SHVV in ZF4 cells, temporal expressions of the mRNAs and cRNA of viral N and G genes were measured by qRT-PCR. For better comparison, the amounts of the mRNAs and cRNA of viral N and G genes at 0 h post-infection were set as 1. It was clear that both the expressions of viral N and G genes mRNAs were significantly up regulated at 3 h post-infection However, the mRNAs and cRNA of viral N and G genes were dramatically decreased during 6–24 h post-infection indicating that SHVV could replicate at the early time point post of infection, but the viral replication was abortive at the late stage of the infection (Figure 2).

### 2.3. Temporal Expression of IFN and Mx in ZF4 after Infection with SHVV

**Figure 3:**
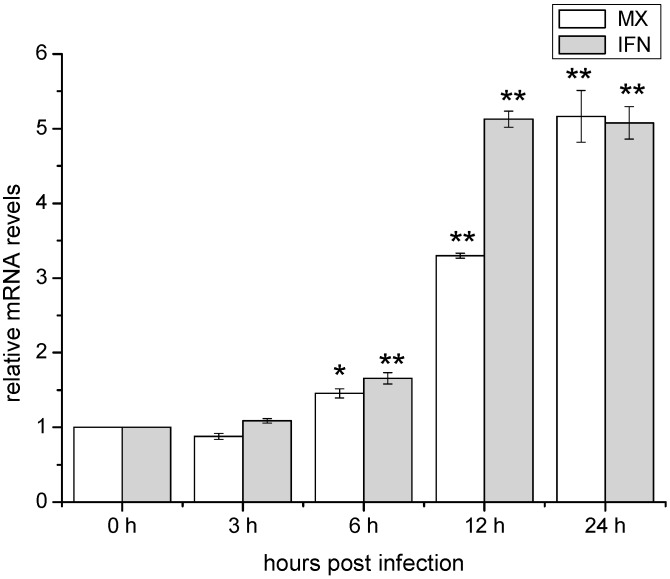
Expression of *Mx* and interferon (*IFN*) genes in ZF4 with time after SHVV infection. Expression was determined by qRT-PCR and β-actin was used as an internal control. Data were shown as mean ± SE (*n* = 3). Fisher’s LSD test was performed. The data were submitted to one-way analysis of variance (one-way ANOVA) followed by Fisher’s LSD test using SPSS. The asterisk indicated a statistically significant difference (** *p* < 0.01, * *p* < 0.05) compared with 0 h (set as 1).

The over expression of IFN has been reported in cells infected with many RNA viruses [3,5,7,16,22,23]. To identify the host factors which might be involved in the inhibition of SHVV replication, the expressions of mRNAs of *IFN* and *Mx* genes were quantified by qRT-PCR. As shown in Figure 3, amounts of the mRNAs of INF and Mx were significantly over expressed during 6–24 h post-infection. The maximum amount of the mRNAs of INF and Mx was observed at 12 and 24 h post-infection respectively. This was agreed with that *Mx* is the downstream gene induced by INF.

### 2.4. Temporal mRNA Expressions of RIG-I, MDA5, DrLGP2T and DrLGP2a in ZF4 Cells after Infection with SHVV

**Figure 4:**
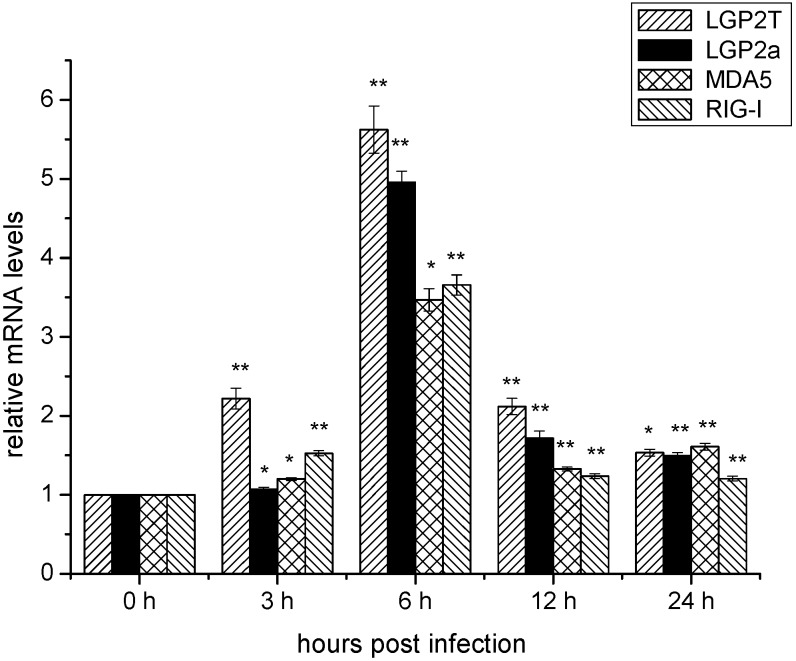
Expression of Retinoic acid-inducible gene I (*RIG-I*), Melanoma differentiation-associated gene 5 (*MDA5*), *LGP2T*, and *LGP2a* genes in ZF4 with time after SHVV infection. Expression was determined by qRT-PCR and β-actin was used as an internal control. Data were shown as mean ± SE (*n* = 3). The data were submitted to one-way analysis of variance (one-way ANOVA) followed by Fisher’s LSD test using SPSS. The asterisk indicated a statistically significant difference (** *p* < 0.01, * *p* < 0.05) compared with 0 h (set as 1).

It has been shown that RLRs pathway has been involved in the activation of IFN expression during some RNA virus replication [3,5,7,16,22,23]. Therefore, the transcripts of the mRNAs of *RIG-I*, *MDA5*, *DrLGP2T* and *DrLGP2a* genes were monitored by qRT-PCR. Since DrLGP2b has a deletion at 525 aa at the *C*-terminus of DrLGP2a, therefore, primers used for DrLGP2b mRNA qRT-PCR could detect the total mRNAs of both DrLGP2 isoforms. However, we could measure the DrLGP2a mRNA alone by qRT-PCR using the pair of specific primers located in the region which was absent in DrLGP2b (Table 1). In the present study, we measured the total amounts of DrLGP2 mRNAs and the amount of LGP2a mRNA, respectively. The results showed that the amounts of the four genes mRNA were all significantly increased at 3 h post-infection, and reached to the peak at 6 h post-infection. Until 24 h post-infection, they were all significantly higher than those at 0 h post-infection, indicating that the expressions of all RLRs genes were activated during the SHVV infection (Figure 4).

### 2.5. Temporal Expressions of DrLGP2a and DrLGP2b Proteins in ZF4 after the Infection with SHVV

**Figure 5:**
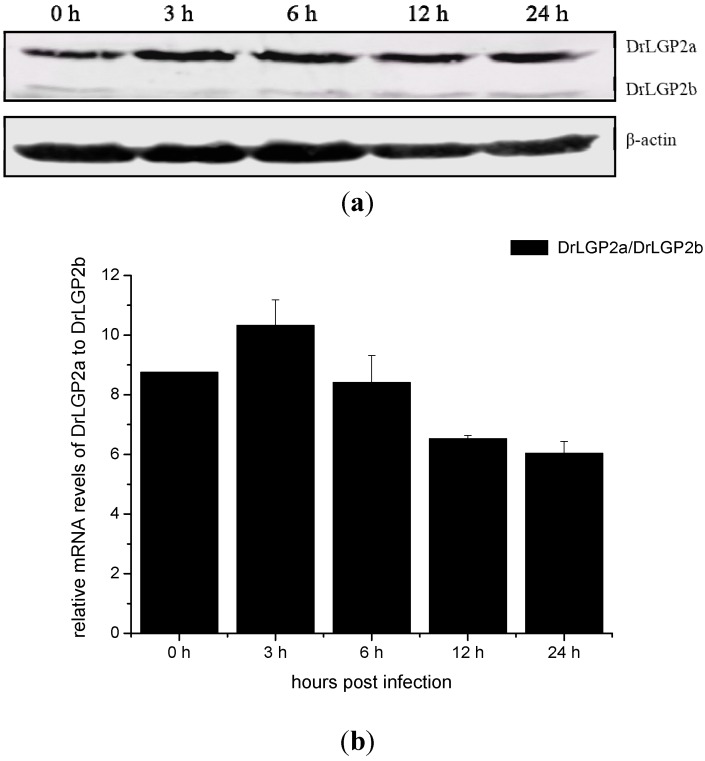

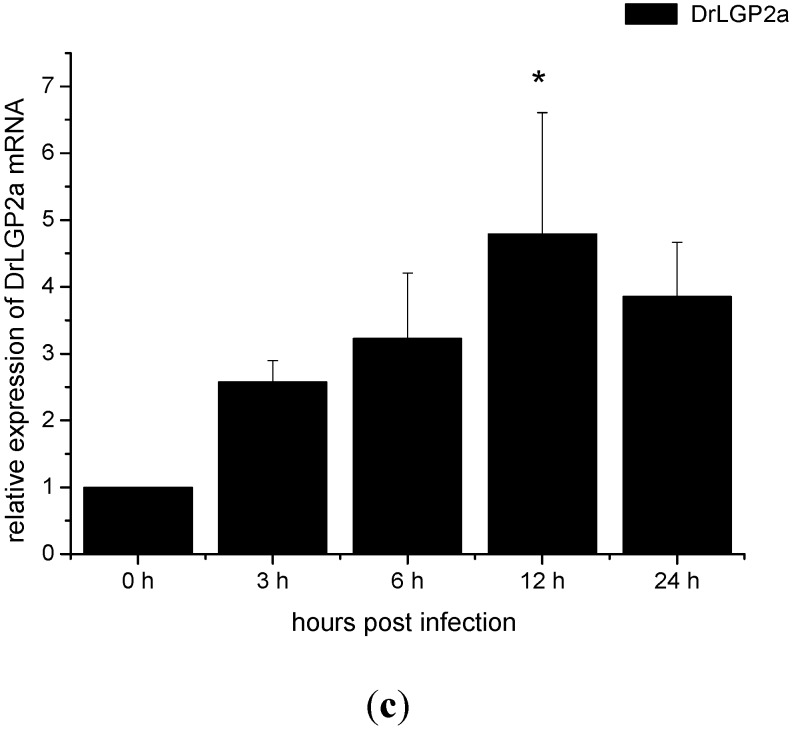
(**a**) The expression of DrLGP2a, DrLGP2b proteins during the infection with SHVV. The samples were harvested at 0, 3, 6, 12, 24 h post-infection and detected by Western blot. Expression of β-actin was used as the loading control .Molecular weight of DrLGP2a, DrLGP2b and β-actin was 77, 55, and 42 kD, respectively; (**b**) The relative expression of DrLGP2a *versus* DrLGP2b proteins infected with SHVV. The samples were harvested at 0, 3, 6, 12, 24 h post-infection and detected by Western blot. Expression of β-actin was used as the internal control. DrLGP2a/DrLGP2b means the amount of DrLGP2a was divided by DrLGP2b. The data were submitted to one-way analysis of variance (one-way ANOVA) followed by Fisher’s LSD test using SPSS. Data were shown as mean ± SE (*n* = 3); and (**c**) The relative expression of DrLGP2a proteins infected with SHVV. The samples were harvested at 0, 3, 6, 12, 24 hpi and detected by Western blot. Expression of β-actin was used as the loading control. Data were shown as mean ± SE (*n* = 3). The data were submitted to one-way analysis of variance (one-way ANOVA) followed by Fisher’s LSD test using SPSS. The asterisk indicated a statistically significant difference (* *p* < 0.05) compared with 0 h (set as 1).

To further analyze the activation of *DrLGP2a* and *DrLGP2b* genes at the protein level, GST fused DrLGP2-DEXDc protein was used for the generation of antibody in rabbit. The titers of the antibody was about 6400 and it was specific for the recognition of DrLGP2-DEXDc domain, since the DrLGP2-DEXDc protein without GST fusion could also be recognized by the antibody. The antibody could recognize both DrLGP2a and DrLGP2b, since the ATP dependent DEXDc domain was mostly located at the 1–220 aa region of the *N*-terminus of the protein. The lysates of ZF4 cells infected with SHVV at different time points were subject to Western blot assay. The results showed that the amount DrLGP2a was about 10 times higher than that of DrLGP2b (Figure 5a,b). Furthermore, DrLGP2a but not DrLGP2b was significantly up regulated at 12 h post-infection (Figure 5c).

**Table 1:** Primers used in the experiments.

Names	Sequence (5'→3')	Application
*LGP2*-FW	CATTAATGAGAAGGGCATCGTT	*DrLGP2* core fragment
*LGP2*-BW	GGCTCAGATTCTGGAAAACGC	*DrLGP2* core fragment
5' RACE Outer Primer	CATGGCTACATGCTGACAGCCTA	*DrLGP2* 5' RACE
5' RACE Inner Primer	CGCGGATCCACAGCCTACTGATGATCAGTCGATG	*DrLGP2* 5' RACE
3' RACE Outer Primer	TACCGTCGTTCCACTAGTGATTT	*DrLGP2* 3' RACE
3' RACE Inner Primer	CGCGGATCCTCCACTAGTGATTTCACTATAGG	*DrLGP2* 3' RACE
*LGP2-DEXDc*-FW	ATCGAATTCATGGAGATCACTCTTAGATCAT	*DrLGP2* antibody
*LGP2-DEXDc*-BW	ATCGCGGCCGCGTGCTGGTCTACCAGGT	*DrLGP2* antibody
*LGP2T*-FW	ATGTGGCGTTACGTGGAGAA	*DrLGP2T* qRT-PCR
*LGP2T*-BW	TCTGCAGCATCGGTGTGTAA	*DrLGP2T* qRT-PCR
*LGP2a*-FW	GGCTGAACGGAAGAGGGAT	*DrLGP2a* qRT-PCR
*LGP2a*-BW	TTCCTGATGTCTCCTCCGC	*DrLGP2a* qRT-PCR
*RIG-I*-FW	TAAGGTGCTGAAGATGGCTC	*RIG-I* qRT-PCR
*RIG-I*-BW	TGATGGTGTTCTGTCCGTTG	*RIG-I* qRT-PCR
*MDA5*-FW	AGCCAGTTATCTGATCGGCG	*MDA5* qRT-PCR
*MDA5*-BW	TCAGCGATGTCCAAACCCTC	*MDA5* qRT-PCR
*IFN*-FW	TGCGTCTACTTGCGAATG	*IFN* qRT-PCR
*IFN*-BW	GGCTTGGAAATGGTGTCT	*IFN* qRT-PCR
*Mx*-FW	CTTGCGTGAGATGAGGTTGC	*Mx* qRT-PCR
*Mx*-BW	TGTCTGGCGGCTCAGTAAGT	*Mx* qRT-PCR
*SHVV-*N-FW	CCGCATCGGAAATCAAGCAG	*N* gene mRNA qRT-PCR
*SHVV-*N-BW	GTTGACCGCTTGCCCAATTT	*N* gene mRNA qRT-PCR
*SHVV-*G-FW	ACACCATACATGCCAGAGGC	*G* gene mRNA qRT-PCR
*SHVV-*G-BW	GCCTCGCTGGGTATCCAAAT	*G* gene mRNA qRT-PCR
*SHVV*-N-FW	CCGCATCGGAAATCAAGCAG	*N* gene cRNA qRT-PCR
*SHVV*-N-BW	GTTGACCGCTTGCCCAATTT	*N* gene cRNA qRT-PCR
*SHVV*-G-FW	ACACCATACATGCCAGAGGC	*G* gene cRNA qRT-PCR
*SHVV*-G-BW	GCCTCGCTGGGTATCCAAAT	*G* gene cRNA qRT-PCR
*β-actin*-FW	CACTGTGCCCATCTACGAG	*β-actin* qRT-PCR
*β-actin*-BW	CCATCTCCTGCTCGAAGTC	*β-actin* qRT-PCR

*LGP2*: Laboratory of genetics and physiology 2; *RIG-I*: Retinoic acid-inducible gene I; *MDA5*: Melanoma differentiation-associated gene 5; *INF*: interferon; *Mx*: interferon-induced GTP-binding protein; *SHVV*: snakehead fish vesiculovirus; N: Nucleoprotein; G: Glycoprotein; FW: forward primer; BW: backward primer.

### 2.6. The Activation of the IFN-α mRNA Expression Was Associated with the Viral Replicative Intermediates

**Figure 6:**
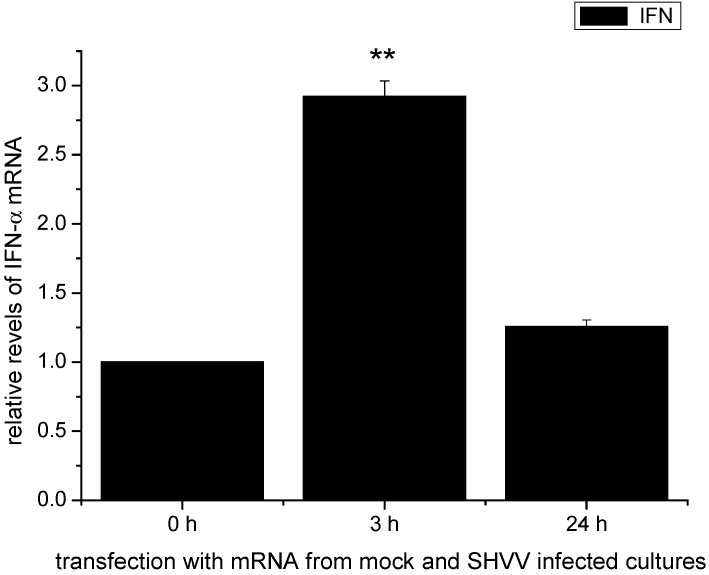
Expression of the INF-α in ZF4 transfected with total RNA from ZF4 cultures either 3 or 24 h after SHVV infection. Data were shown as mean ± SE (*n* = 3). The data were submitted to one-way analysis of variance (one-way ANOVA) followed by Fisher’s LSD test using SPSS. The asterisk indicated a statistically significant difference (** *p* < 0.01) compared with mock (set as 1).

Total RNAs extracted from ZF4 cells infected with or without SHVV at 3 and 24 h post-infection were used to transfect ZF4 cells. Thereafter, total RNAs of the transfected ZF4 cells were extracted and the expression of IFN-α mRNA was quantified by qRT-PCR. As shown in Figure 6, only RNAs from cells infected with SHVV at MOI of 3 was able to significantly increase the expression of *IFN-α* gene. Since significant amounts of viral mRNA and cRNA were detected in ZF4 cells infected with SHVV at 3 h post-infection, but not at 24 h post-infection; therefore, it is reasonable to believe that the viral replicative intermediates were involved in the activation of IFN-α expression.

## 3. Discussion

The outcome of a virus infection is the result of the battle between the host and virus. On one hand, virus tries to highjack the host machinery to facilitate its own replication. On the other hand, host cells employ multiple pathways to limit the multiplication of the virus. Innate immune system is an important defense against viral infections. One of the major mechanisms of innate immune responses is to activate intracellular RLRs and its downstream pathways, leading to the type I interferon (IFN) production and activation of the host antiviral activity. Binding to the viral RNAs is required for the efficient activation of RLRs pathway. For the viral RNA ligands, it is generally believed that RIG-I tends to recognize relative shorter viral RNA template, while MDA5 prefers to relative longer RNA template [21]. Recently, the detail structures of RNA agonists for RIG-I activation have been elucidated using fully chemical synthetic 5'-triphosphate RNAs [24,25,26]. For efficient RIG-I agonist, RNA structure must meet the following three criteria: (1) a triphosphate group (3p-) at the 5' end of the sense strand of the dsRNA; (2) a dsRNA with more than 22 nucleotides; and (3) a blunt 5'- triphosphate end of the dsRNA [24,25,26]. Even though SHVV is a negative single stranded RNA virus, the viral replication can generate a great amount of viral RNAs intermediates, including viral mRNAs, cRNAs and genomic RNAs (gRNA) [27]. Since there is cap structure of viral mRNA, therefore, it cannot activate RLRs pathway. The viral cRNA and genomic RNA are complementary, they form 5'-end blunt double stranded RNAs which are much longer than 22 nt [27]. In addition, there is no cap structure but *tri*-phosphates at the 5' end of the viral cRNA or genomic RNA [27]. Therefore, the viral cRNA-gRNA complex generated during the SHVV infection met the criteria for the efficient RIG-I agonist. In this report, the expressions of mRNAs and cRNAs of the viral N and G genes were significantly expressed at 3 h post-infection, indicating that SHVV could enter the ZF4 cells and initiated the virus replication. However, both mRNAs and cRNAs of the two viral genes were rapidly degraded after 6 h post-infection, suggesting that the host defense has dominated the battle between the host and the virus. To further elucidate the mechanism of the inhibition of virus replication, the expressions of the *IFN* and *Mx* genes were measured, and they were all significantly up regulated after 6 h post-infection, indicating that the virus infection has activated the IFN pathway. Since SHVV is an RNA virus, therefore, the expressions of *RLRs* genes were chosen for further study. The results showed that all mRNAs of the three *RLRs* genes were significantly increased at 3 h post-infection, reached the peak at 6 h post-infection, subsequently they were decreased until 24 h post-infection. However, they were still significantly higher than the basal level (0 h post-infection). Apparently, SHVV infection has activated RIG-I and MDA5-related IFN pathway in ZF4 cells.

It is well established that RIG-I and MDA5 are able to induce cellular immune responses upon recognition of viral dsRNA via CARDs. Since LGP2 lacks CARDs at the *N*-terminal end, it is believed that it is unable to interact with MAVS and hence may serve as a negative mediator for RIGI/MDA5-activated antiviral signaling [22]. However, there is growing evidence that LGP2 is also necessary for effective RIG-I and MDA5-mediated antiviral responses [23]. In mammals, it has been reported that LGP2 played positive regulatory roles in RIG-I/MDA5-activated antiviral signaling in mice infected with encephalomyocarditis virus [22]. Furthermore, ATP dependent DEXDc domain is required for the LGP2-positive regulatory role in mediating RIG-I/MDA5-dependent antiviral responses [23]. It has been reported that there were two isoforms of LGP2 in rainbow trout, named the LGP2a and LGP2b [13]. Furthermore, LGP2a acted as positive regulators of the IFN response. By contrast, the LGP2b antagonized LGP2a function in the rainbow trout cells [13]. In this report, we also identified two LGP2 isoforms from zebrafish, named DrLGP2a and DrLGP2b. The amount of DrLGP2a was about ten times of DrLGP2b protein. Significant over expression of DrLGP2a but not DrLGP2b protein was detected at 12 h post-infection, indicating that DrLGP2a may act as positive regulators of the IFN response during SHVV infection, which was identical with the previous report [13]. The exact roles of the two DrLGP2 isoforms involved in SHVV infection need to be addressed in the future. To further elucidate the mechanism of the activation of RLRs pathway, IFN-α expression in ZF4 cells transfected with total cellular RNAs from ZF4 cells infected with or without SHVV was measured. Only extracted RNAs of ZF4 infected with SHVV at 3 h post-infection were able to activate the expression of IFN, indicating that the viral replicative intermediates were involved in the activation of RLRs. Interestingly another fish Rhabdovirus, Viral Hemorrhagic Septicemia Virus (VHSV), caused abortive infections in a rainbow trout macrophage cell line [28]. Thus the host defense mechanisms described here should be more intensively investigated for this important group of fish viruses.

## 4. Experimental Section

### 4.1. Cell, Virus and Infection

SHVV was isolated and stored in our lab [20]. ZF-4 cells were obtained from Institute of Hydrobiology, Chinese Academy of Sciences (Wuhan, China). ZF4 cells were maintained in DMEM/F12 (1:1) medium (HyClone, Logan, UT, USA) supplemented with 10% fetal bovine serum at 28 °C with 5% CO_2_. They were seeded in six-well plates at a density of 1 × 10^6^ cells per well and were infected with SHVV at multiplicity of infection (MOI) of 1. Thereafter, the infected cells were collected at 0, 3, 6, 12 and 24 h post-infection and used for various assays as described below.

### 4.2. RNA Isolation and cDNA Synthesis

Trizol reagent (TaKaRa, Dalian, China) was used for total cellular RNAs extraction according to the manufacturer’s instructions. The total RNAs were assessed with a NanoDrop Spectrophotometer (NanoDrop Technologies, Wilmington, DE, USA) and were stored at −80 °C until use. cDNAs were synthesized using PrimeScript™ RT reagent Kit with gDNA Eraser (TaKaRa) following the manufacturer’s protocol.

### 4.3. Amplification of Full-Length cDNAs of DrLGP2a and LGP2b by RACE

Since the whole genome sequences of zebrafish have been finished, the predicted zebrafish *LGP2* gene sequences were available. However, there was no report about the full-length cDNA of the *LGP2* genes. Pairs of primers (Table 1) were designed according to the predicted zebrafish *LGP2* gene sequences using Primer Premier 5.0 software (Premier Biosoft International, Palo Alto, CA, USA). Total RNAs were extracted from the ZF4 cells. Subsequently, cDNA synthesis was performed and PCR reactions were carried out in a volume of 25 μL containing 12.5 μL of Premix Ex Taq (TaKaRa), 1 μL of 10 μM of each primer, 9.5 μL of nuclease-free water, and 1 μL of cDNA. Cycling parameters were 94 °C for 3 min followed by 35 cycles of 94 °C for 30 s, 58 °C for 30 s, 72 °C for 2 min; and final extension at 72 °C for 10 min followed by cooling down to 16 °C. The PCR products were resolved by electrophoresis on 1% agarose gels and the fragments of interest were excised, and then purified using the QIAEX II Gel Extraction Kit (Qiagen, Hilden, Germany). The purified fragments were ligated into pMD-19T vectors (Takara) and transformed into *E. coli* DH5α cells according to the standard protocol. Positive clones were screened and sequenced. Based on the obtained DrLGP2 fragment sequences, primers for 5'-RACE and 3'-RACE were designed (Table 1). The RACE reactions were performed by using a 5'-RACE System for Rapid Amplification of cDNA Ends (Invitrogen, Carlsbad, CA, USA) and a SMARTer™ RACE cDNA Amplification Kit (Clontech, Palo Alto, CA, USA) according to the manufacturer’s protocols. The cDNA and deduced amino acid sequences of DrLGP2a and DrLGP2b were analyzed using the BLAST algorithm and the ExPASy (Expert Protein Analysis System) server (University of Geneva, Geneva, Switzerland).

### 4.4. Sequence Analysis

The LGP2 amino acid sequences from various species were obtained from NCBI. The cDNA and deduced amino acid sequence of LGP2 were analyzed using the BLAST algorithm and the ExPASy server (Available online: http://www.expasy.org/tools/). Multiple sequence alignment was performed with the clustalX2 program [29]. A phylogenetic tree was constructed using the neighbour-joining (NJ) method in the Molecular Evolutionary Genetics Analysis (MEGA 4.0) package [30]. Data were analyzed using Poisson’s correction, and gaps were removed by complete deletion. The topological stability of the trees was evaluated by 1000 bootstrap replications.

### 4.5. Generation of Polyclonal DrLGP2-DEXDc Specific Antibody

*N*-terminal fragment of DrLGP2 (1–220 aa) was used for the generation of antibody. Both DrLGP2a and DrLGP2b contained this fragment, which covered the most of the ATP dependent DEXDc domain. Plasmid pGEX-5X-1-DEXDc was constructed and transformed into *E. coli* BL21 (DE3) competent cells (TransGen, Beijing, China). GST fused DrLGP2-DEXDc protein was expressed and purified using Micro Protein PAGE Recovery Kit (Sangon, Shanghai, China) according to the manufacturer’s instructions. The polyclonal antibody specific for DrLGP2-DEXDc was obtained by immunizing rabbit with the purified recombinant protein. The titer of the antibody was determined using an enzyme-linked immunosorbent analysis (ELISA) according to the standard protocol. The antibody specificity was detected by using purified DrLGP2-DEXDc protein without the fusion of GST.

### 4.6. Quantitative Real Time PCR (qRT-PCR) Assay for mRNA Expressions of Target Genes

Primers used for qRT-PCR were listed in Table 1. *β-actin* was used as an internal control. The mRNA expressions of *RIG-I*, *MDA5*, *DrLGP2*, *DrLGP2a*, *IFN*, *Mx*, viral N and G genes were determined by Rotor-Gene Q Series Software 1.7 Real-Time PCR System (Roche Molecular Systems, Branchberg, NJ, USA) in a final volume of 20 μL containing 1 μL cDNA sample, 10 μL SYBR Premix Ex Taq ™ (Takara), 1 μL of each forward and reverse primers and 7 μL nuclease-free water. Cycling parameters were 95 °C for 30 s followed by 40 cycles of 95 °C for 5 s, 58 °C for 20 s, 72 °C for 30 s, finally at 4 °C for 5 min. All reactions were done in triplicate. Dissociation curve analysis was performed after each assay to determine target specificity. The relative expression ratio of the target genes *versus* the *β-actin* gene was calculated using 2^−ΔΔ*C*t^ method, and all data were given in terms of relative mRNA expressed as mean ± SE (*n* = 3). The data were submitted to one-way analysis of variance (one-way ANOVA) followed by Fisher’s LSD test using SPSS 17.0. * *p* < 0.05; ** *p* < 0.01.

### 4.7. Detection Protein Level of DrLGP2 by Western Blot

The total ZF4 lysates were resolved by sodium dodecyl sulfate 10% polyacrylamide gel electrophoresis (SDS-PAGE) and transferred onto nitrocellulose membranes (Bio-Rad, Hercules, CA, USA). Membranes were blocked for nonspecific binding with Odyssey blocking buffer (Li-Cor Biosciences, Lincoln, NE, USA) for 1 h at room temperature and then incubated with a rabbit anti-DrLGP2-DEXDc primary antibody diluted in Odyssey blocking buffer at 1:2000 overnight at 4 °C. Infrared dye-linked goat anti-rabbit IgG antibody (1:15,000) was then added, and membranes were incubated at room temperature for 1 h. β-actin was stable expressed during the infection when it was detected using β-actin specific antibody (Bioss, Beijing, China) and was used as loading control. The results were visualized and quantified using an Odyssey infrared imaging system (Li-Cor Biosciences).

### 4.8. IFN-α Expression in ZF4 Cells Transfected with Total Cellular RNAs

ZF4 cells were infected with or without SHVV at MOI of 1. Cells were harvested at 3 and 24 h post-infection, respectively. Total RNAs of the ZF4 cells were extracted using Trizol (Invitrogen) according to the manufacture’s instruction. 2 μg of the total RNAs were transfected into 2 × 10^6^ ZF4 cells using Amaxa Nucleofector II transfection system (Lonza, Allendale, NJ, USA) under Program T20. Transfected ZF4 cells were maintained in DMEM/F12 (1:1) medium supplemented with 10% fetal bovine serum at 28 °C with 5% CO_2_ for 12 h. Thereafter, total RNAs of the transfected ZF4 cells were extracted as described above. The expression of IFN-α mRNA was quantified by qRT-PCR.

## Supplementary Materials

Supplementary materials can be found at http://www.mdpi.com/1422-0067/16/03/6235/s1.
