# Supplementary material for: Abortive Infection of Snakehead Fish Vesiculovirus in ZF4 Cells Was Associated with the RLRs Pathway Activation by Viral Replicative Intermediates
Source: Int J Mol Sci. 2015 Mar 18;16(3):6235–50. doi: 10.3390/ijms16036235 (PMC4394529; doi:10.3390/ijms16036235)
Supplement: Supplementary file 1 [file ijms-16-06235-s001.pdf]

# Supplementary Information

```
1       ACAAGACCGAAGCAACACACAGACCTCTCGACAGCATACGTATTGCAGAGATGGAGCTCA
1                                                         M  E  L

61      GACTCAGGCCATATCAGGAGGAGGTGGTCCAGGCGGCTTTAAGAGGAGAGAACAGCATCA
4       R  L  R  P  Y  Q  E  E  V  V  Q  A  A  L  R  G  E  N  S  I

121     TCTGGCTGCCCACTGGTGGAGGAAAAACTCGTGCTGCGGTTTACGTCGCTGAGAAACATC
24      I  W  L  P  T  G  G  G  K  T  R  A  A  V  Y  V  A  E  K  H

181     TAGAGACCAAGGCCAATGCCAAAGTGGCAGTGCTCGTCAATAAGGTGCACCTGGTAGACC
44      L  E  T  K  A  N  A  K  V  A  V  L  V  N  K  V  H  L  V  D

241     AGCACTATATGAAAGAATTTGGCCATTACCTAAGGCACAAATACAGGATAAAGGCCATCA
64      Q  H  Y  M  K  E  F  G  H  Y  L  R  H  K  Y  R  I  K  A  I

301     GTGGTGACAGCAGTGAGAAAGACTTTTTTGGGCGTCTTGTTAGAGTCTCAGACTTGGTCA
84      S  G  D  S  S  E  K  D  F  F  G  R  L  V  R  V  S  D  L  V

361     TCTGCACGGCTCAGATTCTGGAAAACGCCTTGAACAACATGGATGAGGACAAGCACGTGG
104     I  C  T  A  Q  I  L  E  N  A  L  N  N  M  D  E  D  K  H  V

421     AGATCACTGATTTTACCCTACTGATCATAGATGAATGTCATCACACAAATAAGGAGAGTG
124     E  I  T  D  F  T  L  L  I  I  D  E  C  H  H  T  N  K  E  S

481     TCTATAACAAGATTATGTGGCGTTACGTGGAGAAGAAAGTGAGAAAAGAAGGCAGACTGC
144     V  Y  N  K  I  M  W  R  Y  V  E  K  K  V  R  K  E  G  R  L

541     CTCAGATTTTGGGCCTCACAGCATCACCTGGTACAGGAGGAAACAAGTCATTGGATAAAG
164     P  Q  I  L  G  L  T  A  S  P  G  T  G  G  N  K  S  L  D  K

601     CTGTTGAACACGTCCTGCAGATCTGTGCCAATCTGGATTCAAAAATTGTGTCCACCAAGA
184     A  V  E  H  V  L  Q  I  C  A  N  L  D  S  K  I  V  S  T  K

661     ATTACACACCGATGCTGCAGAACTTTGTGCCAAAACCCAAAAAGGAATACGACATTGTCG
204     N  Y  T  P  M  L  Q  N  F  V  P  K  P  K  K  E  Y  D  I  V

721     AAAGAAGAGATAAAGATCCATTTGGTGACCACTTGAAGTCAATGATGTTAATGATTCATG
224     E  R  R  D  K  D  P  F  G  D  H  L  K  S  M  M  L  M  I  H

781     AGTTTATGCCAGCAACGGTGGGTAGCAGCCTGAGAGAACTAGGCACCCAGGAATATGAGG
244     E  F  M  P  A  T  V  G  S  S  L  R  E  L  G  T  Q  E  Y  E

841     CTGATGTGGTGGAACTGGAGAAAGCAGGTGTAAAAGTAAAGAACAGACTGATCGCTCAGT
264     A  D  V  V  E  L  E  K  A  G  V  K  V  K  N  R  L  I  A  Q

901     GCGCTTTGCATCTACGCAAATACAATGATGCCCTTCTCATTAATGACACCATTCGCATGG
```

**Figure S1.** *Cont.*

```
284    C A L H L R K Y N D A L L I N D T I R M

961    TGGATGCGTTCAGAGTGCTGGAAGAGTTTTACAATACAAGAAGCAGCAAGTTGCTGGATG
304    V D A F R V L E E F Y N T R S S K L L D

1021   GAACAGACATCTTTCTACAAGGACTTTTCGATGAGAACAGTCTGGAGTTAAAACATCTGG
324    G T D I F L Q G L F D E N S L E L K H L

1081   CGTCGGACGCCCGATATGAAAACCCCAAACTAGCTCAACTGCAGAGCAGATTGCTGGAAG
344    A S D A R Y E N P K L A Q L Q S R L L E

1141   AGTTCCAAGACACTAATTCTCGTGGAATCATCTTCTCAAAGACACGCAGAGGCACGCATT
364    E F Q D T N S R G I I F S K T R R G T H

1201   GTCTGAATGACTGGGTGAAAACAAACCGTGAGCTGCAGAGGGTCAACATCACTGCTGGCA
384    C L N D W V K T N R E L Q R V N I T A G

1261   TTCTGACCGGAGCCGGTAATGGCGCTAACAACATGACACAGACTGAACAGAAGAGCGTCA
404    I L T G A G N G A N N M T Q T E Q K S V

1321   TCAGTCATTTTAGGCAAGGATACCTCAACCTCCTCATCTCCACCAGTGTAGCTGAGGAGG
424    I S H F R Q G Y L N L L I S T S V A E E

1381   GACTGGATATTCCTGAATGCAACTTAGTTGTGCGTTACGGGCTGTTGACCAATGAAATCG
444    G L D I P E C N L V V R Y G L L T N E I

1441   CTCAGCAGCAGGCCAGCGGACGAGCTCGAGCCTCCAACAGTGTCTACTCTGTGGTGGCTG
464    A Q Q Q A S G R A R A S N S V Y S V V A

1501   ACGTAGGTGGACGGGAAGTGCGCAAAGAACTTGTCAATGAGTATCTAGAAGATCTCACCG
484    D V G G R E V R K E L V N E Y L E D L T

1561   CAAGAGCTATTGATGAAGTACAGCGCATGAGTCCTGTGGACTTCAGACACAAGGTGTTTG
504    A R A I D E V Q R M S P V D F R H K V F

1621   AGCTCCAGAAAACAGCTGTGGTGATTCGGATGGAGGCTGAACGGAAGAGGGATGCGAAAA
524    E L Q K T A V V I R M E A E R K R D A K

1681   AGCAGCGCTATAGTCCAGGTCAGGTTCAGCTTCAGTGCAGAAGCTGCTTCGCTTCCGTCT
544    K Q R Y S P G Q V Q L Q C R S C F A S V

1741   GCAGCGGAGGAGACATCAGGAAGATCGAGAACTCGCACCATGTCAACGTTAACACTGAGT
564    C S G G D I R K I E N S H H V N V N T E

1801   TTAAAAATCACTATAAAGTTGGTGGCCAGGTGAATATGGAGAGGACCTTTGAAGACTGGG
584    F K N H Y K V G G Q V N M E R T F E D W
```

**Figure S1.** *Cont.*

```
1861    AGCCTGGACGGATTATCAGCTGCAGACGTTGTGGAGAGGACTGGGGATTCGAGATTAAAT
604     E  P  G  R  I  I  S  C  R  R  C  G  E  D  W  G  F  E  I  K

1921    TCAAGAAAGTGGCAATTCTCCCCTGTCTGAACATAAAGAGCTTCTCCTTTAACACCCCTA
624     F  K  K  V  A  I  L  P  C  L  N  I  K  S  F  S  F  N  T  P

1981    AAGAAACAAAGCCTTACAAGAAGTGGAAGGATGTTGAGTTTCAGGTGACAGAGTTTGACT
644     K  E  T  K  P  Y  K  K  W  K  D  V  E  F  Q  V  T  E  F  D

2041    TTATTGAGTACATGAGCTGCCGTTTCCCTGACCTGGACTTGTCTGACTGATACAGCTCCA
664     F  I  E  Y  M  S  C  R  F  P  D  L  D  L  S  D  *

2101    TACCACTGATTTAATTGAAATATGAAATAATACATATTCTGATTATGTTAATGCTCCGTC
2161    TTGGGTCTTCATGCATGTTGGTGACAGTAGTAGAAGCAGATAGATGGTTTTATTCAAAAC
2221    TAGGGCTGTCAATTGATTTAAAAAATTAACTAAGTAATCACAGTTTTTGTAATTAATCA
2281    TAATTAATCATGGAATGCCATGTTTATTACAGAAATAGAAACACAGGCATGTAAGTGCCA
2341    TTTGAATTTCAAAATAATCAATGCCAATAACAAACAAAAACATTTCCATGTTGGATTCTA
2401    ATTGGACTACAAAAAAATCCAAAATACAGGCATTGCAAATATAGAAAAAAAAAAAAAAAA
2461    AAAAAAAAAAAA
```

**Figure S1.** Nucleotide and deduced amino acid sequences of DrLGP2a from *Danio rerio*. The start codon (ATG) was shown by underline. The termination code (TAG) was marked with an asterisk below. The motif associated with mRNA instability (ATTTA) was in bold underline. Shadow said DEXDc (DEAD/DEAH box helicase domain), double underline said HELICc, the box said RD (regulatory domin).

```
1       ACAAGACCGAAGCAACACACAGACCTCTCGACAGCATACGTATTGCAGAGATGGAGCTCA
1                                                         M  E  L

61      GACTCAGGCCATATCAGGAGGAGGTGGTCCAGGCGGCTTTAAGAGGAGAGAACAGCATCA
4       R  L  R  P  Y  Q  E  E  V  V  Q  A  A  L  R  G  E  N  S  I

121     TCTGGCTGCCCACTGGTGGAGGAAAAACTCGTGCTGCGGTTTACGTCGCTGAGAAACATC
24      I  W  L  P  T  G  G  G  K  T  R  A  A  V  Y  V  A  E  K  H

181     TAGAGACCAAGGCCAATGCCAAAGTGGCAGTGCTCGTCAATAAGGTGCACCTGGTAGACC
44      L  E  T  K  A  N  A  K  V  A  V  L  V  N  K  V  H  L  V  D

241     AGCACTATATGAAAGAATTTGGCCATTACCTAAGGCACAAATACAGGATAAAGGCCATCA
64      Q  H  Y  M  K  E  F  G  H  Y  L  R  H  K  Y  R  I  K  A  I

301     GTGGTGACAGCAGTGAGAAAGACTTTTTGGGCGTCTTGTTAGAGTCTCAGACTTGGTCA
84      S  G  D  S  S  E  K  D  F  F  G  R  L  V  R  V  S  D  L  V

361     TCTGCACGGCTCAGATTCTGGAAAACGCCTTGAACAACATGGATGAGGACAAGCACGTGG
104     I  C  T  A  Q  I  L  E  N  A  L  N  N  M  D  E  D  K  H  V
```

**Figure S2.** *Cont.*

```
421     AGATCACTGATTTTACCCTACTGATCATAGATGAATGTCATCACACAAATAAGGAGAGTG
124     E  I  T  D  F  T  L  L  I  I  D  E  C  H  H  T  N  K  E  S

481     TCTATAACAAGATTATGTGGCGTTACGTGGAGAAGAAAGTGAGAAAAGAAGGCAGACTGC
144     V  Y  N  K  I  M  W  R  Y  V  E  K  K  V  R  K  E  G  R  L

541     CTCAGATTTTGGGCCTCACAGCATCACCTGGTACAGGAGGAAACAAGTCATTGGATAAAG
164     P  Q  I  L  G  L  T  A  S  P  G  T  G  G  N  K  S  L  D  K

601     CTGTTGAACACGTCCTGCAGATCTGTGCCAATCTGGATTCAAAAATTGTGTCCACCAAGA
184     A  V  E  H  V  L  Q  I  C  A  N  L  D  S  K  I  V  S  T  K

661     ATTACACACCGATGCTGCAGAACTTTGTGCCAAAACCCAAAAAGGAATACGACATTGTCG
204     N  Y  T  P  M  L  Q  N  F  V  P  K  P  K  K  E  Y  D  I  V

721     AAAGAAGAGATAAAGATCCATTTGGTGACCACTTGAAGTCAATGATGTTAATGATTCATG
224     E  R  R  D  K  D  P  F  G  D  H  L  K  S  M  M  L  M  I  H

781     AGTTTATGCCAGCAACGGTGGGTAGCAGCCTGAGAGAACTAGGCACCCAGGAATATGAGG
244     E  F  M  P  A  T  V  G  S  S  L  R  E  L  G  T  Q  E  Y  E

841     CTGATGTGGTGGAACTGGAGAAAGCAGGTGTAAAAGTAAAGAACAGACTGATCGCTCAGT
264     A  D  V  V  E  L  E  K  A  G  V  K  V  K  N  R  L  I  A  Q

901     GCGCTTTGCATCTACGCAAATACAATGATGCCCTTCTCATTAATGACACCATTCGCATGG
284     C  A  L  H  L  R  K  Y  N  D  A  L  L  I  N  D  T  I  R  M

961     TGGATGCGTTCAGAGTGCTGGAAGAGTTTTACAATACAAGAAGCAGCAAGTTGCTGGATG
304     V  D  A  F  R  V  L  E  E  F  Y  N  T  R  S  S  K  L  L  D

1021    GAACAGACATCTTTCTACAAGGACTTTTCGATGAGAACAGTCTGGAGTTAAAACATCTGG
324     G  T  D  I  F  L  Q  G  L  F  D  E  N  S  L  E  L  K  H  L

1081    CGTCGGACGCCCGATATGAAAACCCCAAACTAGCTCAACTGCAGAGCAGATTGCTGGAAG
344     A  S  D  A  R  Y  E  N  P  K  L  A  Q  L  Q  S  R  L  L  E

1141    AGTTCCAAGACACTAATTCTCGTGGAATCATCTTCTCAAAGACACGCAGAGGCACGCATT
364     E  F  Q  D  T  N  S  R  G  I  I  F  S  K  T  R  R  G  T  H

1201    GTCTGAATGACTGGGTGAAAACAAACCGTGAGCTGCAGAGGGTCAACATCACTGCTGGCA
384     C  L  N  D  W  V  K  T  N  R  E  L  Q  R  V  N  I  T  A  G

1261    TTCTGACCGGAGCCGGTAATGGCGCTAACAACATGACACAGACTGAACAGAAGAGCGTCA
404     I  L  T  G  A  G  N  G  A  N  N  M  T  Q  T  E  Q  K  S  V

1321    TCAGTCATTTTAGGCAAGGATACCTCAACCTCCTCATCTCCACCAGTGTAGCTGAGGAGG
```

**Figure S2.** *Cont.*

424 I S H F R Q G Y L N L L I S T S V A E E

1381 GACTGGATATTCCTGAATGCAACTTAGTTGTGCGTTACGGGCTGTTGACCAATGAAATCG
444 G L D I P E C N L V V R Y G L L T N E I

1441 CTCAGCAGCAGGCCAGCGGACGAGCTCGAGCCTCCAACAGTGTCTACTCTGTGGTGGCTG
464 A Q Q Q A S G R A R A S N S V Y S V V A

1501 ACGTAGGTGGACGGGAAGTGCGCAAAGAACTTGTCAATGAGTATCTAGAAGATCTCACCG
484 D V G G R E V R K E L V N E Y L E D L T

1561 CAAGAGCTATTGATGAAGTACAGCGCATGAGTCCTGTGGACTTCAGACACGAGGTTTGTG
504 A R A I D E V Q R M S P V D F R H E V C

1621 TTTGACTTTTTACTATTAAATCTGACTGTGTTTGTAATAGAATAGAAAAACTTAAAAGGA
524 V *

1681 CAATTCACCTAGACTTCACAATTCTGTCATCATTTACCCAAACATGAGTTTCTTTCTTCT
1741 GTTGAACACAAAAAGAAGTTACTTTTGAATAGTGTTGGAAGCCTGTAACCATTGACTTCC
1801 ATAGTATTTGTTTTTTCTACCATTTAAGTCGGTGGTTACCGGTTTCTAACATACTTCAGA
1861 ATATCCATTTTGTGTTTAACAGAAAAGAGAAACTTCTTAAGGTTTGGAATCACGTGAGTA
1921 AACAGTGAGTACATTTTCATTTTTGAGTAAACTATATTTTTAAAGGTGTTTCACATACGA
1981 AATCTACTGTAAGTCTGTTGACAGATTAGAGTTCTTTGTTAGTATTGCTCCCCAAGTCTA
2041 GGCGAAAACAACATGGGCAT**AATAAA**CAAAGAAAACAAATGTGTTAGGGTGGTGGATTTC
2101 CCAAACAAAACAAAAACTTAATGCAAAAGATCCCTTTCGGTCGACAACCAAACTCGGACA
2161 CTCAGTTGAGAGTATTTAAATAGATTTATTATAGGAAAAAGTAGTAAATATAAATGAGCA
2221 TCCCTTCAGGATCTCCACAGGCCAAAAAAAAAAAAAAAAAAAAAA

**Figure S2.** Nucleotide and deduced amino acid sequences of DrLGP2b from *Danio rerio*. The start codon (ATG) was shown by underline. The termination code (TAG) was marked with an asterisk. The typical polyadenylation signal AATAAA was bolded. The motif associated with mRNA instability (ATTTA) was in bold underline. Shadow said DEXDc (DEAD/DEAH box helicase domain), double underline said HELICc domain structure.

```
DrLGP2a    -MELRLRPYQEEVVQAALRGENSIIWLPTGGGKTRAAVYVAEKHLETKANAKVAVLVNKV
DrLGP2a    -MELRLRPYQEEVVQAALRGENSIIWLPTGGGKTRAAVYVAEKHLETKANAKVAVLVNKV
CiLGP2     -MEITLRSYQEEVVQAALKGENSIIWLPTGGGKTRAAVYVAKKHLETTRNAKVAVLVNKV
IpLGP2     -MEISLRPYQQEVVQVALRGENSIIWLPTGGGKTRAAVYVAKRHLETHPNGKVAVLVNKV
PoLGP2     MAELGLYSYQQEVVERAIKGENVIIWLPTGGGKTRAAVYVAKRHLETTQHAKVVVLVNKV
XlLGP2     ---MELHDYQWEVIGPALEGKNIIIWLPTGAGKTRAALYVAMRHLEMKRNAKVCLMVNKV
GaLGP2     ---MELHGYQLEAVAPALRGRNSIVWLPTGAGKTRAAVHVCRRHLEGRRGGRVAVLVNKV
MmLGP2     ---MELRPYQWEVILPALEGKNIIIWLPTGAGKTRAAAFVAKRHLETVDRGKVVVLVNRV
HsLGP2     ---MELRSYQWEVIMPALEGKNIIIWLPTGAGKTRAAAYVAKRHLETVDGAKVVVLVNRV
              : *  ** *.:  *:.*.* *:*****.****** .*.  :***    .:* ::**:*
```

**Figure S3.** *Cont.*

```
DrLGP2a     HLVDQHYMKEFGHYLRHKYRIKAISGDSSEKDFFGRLVRVSDLVICTAQILENALNNMDE
DrLGP2b     HLVDQHYMKEFGHYLRHKYRIKAISGDSSEKDFFGRLVRVSDLVICTAQILENALNNMDE
CiLGP2      HLVDQHFNNEFNPYLGKAYRVKAISGDSSDKDLFGRLVKDSDLIICTAQILENALNSSEE
IpLGP2      HLVDQHFQKEFRPHLGSSVKMVPISGDSNEKDFFGCVVRDSSLVICTAQILENALINTEE
PoLGP2      HLVDQHYTKEFKPHLGSNYTLVPVSGESELKDLFGKVVQDSDVVICTAQILYNALTNSEE
XlLGP2      HLVDQHFSNEFHPHLKDKYKVVAISGDTEHKCFFAELVQNNDVIICTAQILQNALSSSSE
GaLGP2      HLVQQHLEKEFH-VLRDAFKVTAVSGDSSHKCFFGQLAKGSDVVICTAQILQNALLSGEE
MmLGP2      HLVSQHAE-EFRRMLDKHWTVTTLSGDMGSRAGFGLMARSHDLLICTAELLQLALNSSEE
HsLGP2      HLVTQHGE-EFRRMLDGRWTVTTLSGDMGPRAGFGHLARCHDLLICTAELLQMALTSPEE
            *** **   **   *     : .:**:   :  *. :.:  .::****::*  ** . .*

DrLGP2a     DKHVEITDFTLLIIDECHHTNKESVYNKIMWRYVEKKVRKEGRLPQILGLTASPGTGGNK
DrLGP2b     DKHVEITDFTLLIIDECHHTNKESVYNKIMWRYVEKKVRKEGRLPQILGLTASPGTGGNK
CiLGP2      EKHVELTDFSLLIIDECHHTQKESVYNKIMGRYVEKKVRKERKLPQVLGLTASPGTGGNK
IpLGP2      SKHVELTDFTLLIIDECHHTHKEGVYNKIMARYVRKKIERVKGLPQILGLTASPGTGGAK
PoLGP2      TKHVELSDITLLIFDECHHTHKGGVYNQIMKCYVEKKLKGERRLPQILGLTASPGTGGAR
XlLGP2      EIHVELTDFTLLIIDECHHTHKDGVYNKLMEGYLERKITQKGKLPQILGLTASPGTGRAT
GaLGP2      EARVELTDFSLLVIDECHHTQKEAVYNKIMLSYLQKKLSGQRDLPQILGLTASPGTGGET
MmLGP2      DEHVELREFSLIVVDECHHTHKDTVYNTILSRYLEQKLKKAEPLPQVLGLTASPGTGGAT
HsLGP2      EEHVELTVFSLIVVDECHHTHKDTVYNVIMSQYLELKLQRAQPLPQVLGLTASPGTGGAS
              :**:  ::*::.******:*  *** ::  *:. *:     ***:**********

DrLGP2a     SLDKAVEHVLQICANLDS-KIVSTKNYTPMLQNFVPKPKKEYDIVERRDKDPFGDHLKSM
DrLGP2b     SLDKAVEHVLQICANLDS-KIVSTKNYTPMLQNFVPKPKKEYDIVERRDKDPFGDHLKSM
CiLGP2      TLAKAVEHVLQICANLDS-VIVSTKNYASKLKEVVPRPRKQYDIVEKRALDPFGDHLKLM
IpLGP2      SLDGAITHVLEICANLDS-VIVSTKNYEAELKEAVPRPMKNYDIVDERYWDPFGDHLKMM
PoLGP2      ILEKAVEHVLQICANLDS-SIVSTRLYAPELKKKVPRPIKTFDIVENRPEDPFGNHLKRM
XlLGP2      SFEKAEEHILQICANLDTWRIMSAEVHREDLEAKAKQPNKQYDLVTERPRDPFGDKLKEL
GaLGP2      SFEGAVEHILQICANLDTEVIASAQEHAQHLQSHVPQPTKQYDLCQEREQDPFGQRLKKI
MmLGP2      KLQGAIDHILQLCANLDTCHIMSPKNCYSQLLMHNPKPCKQYDLCQRRAQDPFGDLIKKL
HsLGP2      KLDGAINHVLQLCANLDTWCIMSPQNCCPQLQEHSQQPCKQYNLCHRRSQDPFGDLLKKL
             :  *  *:*::*****:  * *..     *     :* * :::  .*  ****: :* :

DrLGP2a     MLMIHEFMPATVG-SSLRELGTQEYEADVVELEKAGVKVKNRLIAQCALHLRKYNDALLI
DrLGP2b     MLMIHEFMPATVG-SSLRELGTQEYEADVVELEKAGVKVKNRLIAQCALHLRKYNDALLI
CiLGP2      MSLIHEFMPSTVS-RSLREVGTQDYEADVVELEKTGVKEENRLITQCALHLRQYNDALLI
IpLGP2      MKLIHEFMMLDRK-FTLREMGTQEYEADVVELEKKGVQEQNRLLARCALHLRKFNDALLI
PoLGP2      MQMIQDYMNLPTSDFRLRECGTQEYEADVVILEQRGIREDNRRLAQCALHLRQYNDALLI
XlLGP2      MKTIHEYLRTTDF--CESDFGTQLYEQKVVELEKEGAVEANRMKRTCALHLRKYNDSLLV
GaLGP2      MAQIQEHMEMPEL--PQN-FGTQVYEQRIVELENRAAERFCRKTRVCALHLRRYNDALLI
MmLGP2      MNQIHQQLEMPDL--KQQ-FGTQMYEQQVVQLCKDAAEAGLQEQRVYALHLRRYNDALFI
HsLGP2      MDQIHDHLEMPEL--SRK-FGTQMYEQQVVKLSEAAALAGLQEQRVYALHLRRYNDALLI
            *  *:: :            *** **  :* * : .     :     *****::**:*::

DrLGP2a     NDTIRMVDAFRVLEEFYNTR--SSKLLDGTDIFLQGLFDENSLELKHLASDARYENPKLA
DrLGP2b     NDTIRMVDAFRVLEEFYNTR--SSKLLDGTDIFLQGLFDENSLELKHLASDARYENPKLA
```

**Figure S3.** *Cont.*

```
CiLGP2      HDTVRMVDAFNVLDEFYNSR--SNKLLDGTDFFLQGLFDENRVELKQLALDARYENPKLA
IpLGP2      NDTVRMVDALRLLEEFYTTE--TRNVLDITDEFLTGLFNENKVELLQLASEASNENPKLE
PoLGP2      NDTLLMMDAYRNLEEFYISK--STRAIDKTDFFLVGIFQDNQADLMKVARDSRYENPKMA
XlLGP2      HDTVRMMDAYELLDDYYQQEKVIRKQNDPTDAFLIQLFDGNRARLLELAQDVRFENPKLR
Gallus      NDTVRMMDAFQCLQQFYADK---RDTKDPTERFLATTFEENRATLQALAGDQRYENPRLS
MmLGP2      HDTVRARDALDMLQDFYDRERTTKTQMVRAESWLLKLFDDHKNVLGQLAAR-GPENPKLE
HsLGP2      HDTVRAVDALAALQDFYHREHVTKTQILCAERRLLALFDDRKNELAHLATH-GPENPKLE
            :**:   **   *:::*  .          ::  *   *: .    *  :*     ***::

DrLGP2a     QLQSRLLEEFQDT-NSRGIIFSKTRRGTHCLNDWVKTNRELQRVNITAGILTGAG--NGA
DrLGP2b     QLQSRLLEEFQDT-NSRGIIFSKTRRGTHCLNDWVKTNRELQRVNITAGILTGAG--NGA
CiLGP2      QLQRTLLKEFKENKMSRGIIFSKTRRGTHCLYDWVNANHELQKVKISAGILTGTG--TGV
IpLGP2      RLQRTLVDQFKDE-NSRGIIFAKTREITRCLYDWVRTNPELRRANIRAANLVGAG--TGA
PoLGP2      KLESVLLKQFSSGLQSKGILFSKTRKSIHCLNEWVLNNRALQEAGVKSAILTGAG--NGI
XlLGP2      KLEEILRDQFQFSSGSRGIIFTRTRQSTHSLHNWISSKHSFQIMGVKTAPLTGAGYSNQS
GaLGP2      KLEEILQEHFQPPGSSRGIVFTKTRQSAHSLLSWLQDTAGLCGQHIRAAVLTGSGHSNQA
MmLGP2      MLERILLKQFGSPGHTRGIIFTRTRQTASSLLLWLRQQPCLQTVGIKPQMLIGAGNTSQS
HsLGP2      MLEKILQRQFSSSNSPRGIIFTRTRQSAHSLLLWLQQQQGLQTVDIRAQLLIGAGNSSQS
             *:  *  .*      .:**:*::**.   .*  *:      :    : .  * *:*  .

DrLGP2a     NNMTQTEQKSVISHFRQGYLNLLISTSVAEEGLDIPECNLVVRYGLLTNEIAQQQASGRA
DrLGP2b     NNMTQTEQKSVISHFRQGYLNLLISTSVAEEGLDIPECNLVVRYGLLTNEIAQQQASGRA
CiLGP2      NHMNQNKQKNTIKDFRLGHLNLLISTSVAEEGLDIAECNLVVRYGLLTNEIAQQQASGRA
IpLGP2      THMTQREQRDTIKTFREGELNLLISTSVAEEGLDIPECNVVIRYGLLTNEIAQQQASGRA
PoLGP2      TYMTQHEQADTICRFRQGTLNLLISTSVAEEGLDIPECNLVVRYGLLTNEIAQQQASGRA
XlLGP2      KHMTQNEQRETIEMFRKGQLNLLISTSVAEEGLDIPQCNIVVRYGLMTNEISMVQARGRA
GaLGP2      KGMTQNEQQDVITLFRYGELNLLFSTSVAEEGLDIPECNIVVRYGLMTNEIAMVQAQGRA
MmLGP2      THMTQKDQQEVIQEFRDGILSLLVATSVAEEGLDIAQCNVVVRYGLLTNEISMVQARGRA
HsLGP2      THMTQRDQQEVIQKFQDGTLNLLVATSVAEEGLDIPHCNVVVRYGLLTNEISMVQARGRA
            . *.* .* ..*  *: * *.**.:**********..**:*:****:****:  ** ***

DrLGP2a     RASNSVYSVVADVGGREVRKELVNEYLEDLTARAIDEVQRMSPVDFRHKVFELQKTAVVI
DrLGP2b     RASNSVYSVVADVGGREVRKELVNEYLEDLTARAIDEVQRMSPVDFRHEVCV--------
CiLGP2      RALNSVYSVVAEKGGRELRREFTNEYLESLTSNAIDQVQSMSPREFRIKITELQQMAVLI
IpLGP2      RAKNSVYSVVAKAGGREMRKEKTNEYLEELTSKAIAHVQSMEPGRFRQKIFELQHEAVTA
PoLGP2      RAQDSQYSVVALKGGREERREITNEYLEELTAKAIAQVQDMSIREFQRKITELQTQAVIK
XlLGP2      RHEDSCYSFLAKIGGKEIRREETNETLEGLMKRAIEAVQRMPEQEYQKKIKELQEESVIA
GaLGP2      RAQNSMYSVLAKANSREVYREQLNESLVGLMERAIRAVQAMPERKYRLKIVELQRNAVLS
MmLGP2      RAGQSVYSFLATEGSREMKRELTNEALEVLMEKAVAAVQKMDPDEFKAKIRDLQQASLVK
HsLGP2      RADQSVYAFVATEGSRELKRELINEALETLMEQAVAAVQKMDQAEYQAKIRDLQQAALTK
            *  :* *:.:*  ..:*  :*  ** *  *  .*:  ** *    :: ::

DrLGP2a     RMEAERKRDAKKQRYSPGQVQLQCRSCFASVCSGGDIRKIENSHHVNVNTEFKNHYKVG-
DrLGP2b     ------------------------------------------------------------
CiLGP2      RIEAERKKDARKQRYSPDQVHLQCRGCFVFVGLGEDIRKIENSHHVNINPEFQRHYRTG-
IpLGP2      RILMANQRERRRLRYDAAEVEFDCRGCFTAVARGDDMRLVNDTQHININPDFERFYKVG-
PoLGP2      SRIEESCKMQKRSRYTASSIQLLCRNCFKSVASGSDIKLIDNVHHVNVNPAFEKHYKVG-
```

**Figure S3.** *Cont.*

```
XlLGP2      RKVKQAKRDQKRNTFYPEQVRFYCRCCSQAVAHGDDFRTIEGTHYVNINSDFRIYYEVCS
GaLGP2      WQVKEARSSERRQLHDPDDVYFHCVNCNVAVCRGSDIRTVEAMHHVNINPNFRFYYTVSS
MmLGP2      RAARAAHREIQQGQFLPEHVQLLCINCMVAVGYGSDLRKVEGTHHVNVNPNFSVYYTTSQ
HsLGP2      RAAQAAQRENQRQQFPVEHVQLLCINCMVAVGHGSDLRKVEGTHHVNVNPNFSNYYNVSR

DrLGP2a     GQVNMERTFEDWEPGRIISCR--RCGEDWGFEIKFKKVAILPCLNIKSFSFNTPKETKPY
DrLGP2b     ------------------------------------------------------------
CiLGP2      KQVFLEKTFEDWEPGQVINCR--KCGKDWGMEIKFKKVAILPCLNIKSFSLDTPGGKFTH
IpLGP2      GQVYLERTFEDWEPGRTISCV--ACGQNWGMEVKLKN-LVLPCLNIKGFSMKTRQSSRTA
PoLGP2      GQVILPKMFDDWEPGRIISCNNGNCNKEWGFEMKYKEIALLPNLAIKHLFLQTPDGRMTV
XlLGP2      PPLDFGKKMVDWTPGGKIRC---LCGQDWGFEMIYKH-VNFPAISVKNFVVETPEIKRPY
GaLGP2      GKIHFERTFRDWEPGCRIVCS--ECRQEWGMEMIYRN-VTLPILSIKNFVVVTPDEKKKY
MmLGP2      NPVVINKVFKDWRPGGTIRCS--NCGEVWGFQMIYKS-VTLPVLKIGSMLLETPRGKIQA
HsLGP2      DPVVINKVFKDWKPGGVISCR--NCGEVWGLQMIYKS-VKLPVLKVRSMLLETPQGRIQA

DrLGP2a     KKWKDVEFQVTEFDFIEYMSCRFPDLDLSD-
DrLGP2b     -------------------------------
CiLGP2      KQWKDVEFQVTEFDFIDYMSSRFPDLDLDD-
IpLGP2      KQWKDIEFLVEEFDFVQYVKNRYPDLEQD--
PoLGP2      KKWKDVPFTVDNFSFEEYCQDNFPDLLD---
XlLGP2      ARWKDVPFPVDELNYVQHVRIHPELLVHFED
GaLGP2      KKWSTVTFPIEEFSYLEYCSSTQDESL----
MmLGP2      KKWSRVPFSIPVFDILQDCTQSLSELSLD--
HsLGP2      KKWSRVPFSVPDFDFLQHCAENLSDLSLD—
```

**Figure S3.** Multiple alignment of LGP2 using the Clustal X2 program. LGP2 are conserved in vertebrates. Identical amino acids among all sequences are indicated by asterisks whereas those with high or low similarity are indicated by ":" and "." respectively. Marked conserved domains include the DEXDc (DEAD/DEAH box helicase domain) (Shadowed), the HELICc (bold underlined) and the RD (regulatory domin) (double underlined). CiLGP2 (*Ctenopharyngodon idella*, AFQ93565.1), Gg (*Gallus gallus*, AEK21509.1), HsLGP2 (*Homo sapiens*, NM_024119.2), IpLGP2 (*Ictaluruspunctatus*, AFS34610.1), MmLGP2 (*Mus musculus*, NM_030150.2), PoLGP2 (*Paralichthys olivaceus*, ADM18136.1), XlLGP2 (*Xenopus laevis*, NP_001085915.1).
